# Knowledge based identification of essential signaling from genome-scale siRNA experiments

**DOI:** 10.1186/1752-0509-3-80

**Published:** 2009-08-05

**Authors:** Armand Bankhead, Iliana Sach, Chester Ni, Nolwenn LeMeur, Mark Kruger, Marc Ferrer, Robert Gentleman, Carol Rohl

**Affiliations:** 1 Rosetta Inpharmatics LLC, a wholly owned subsidiary of Merck & Co., Inc., 401 Terry Ave N, Seattle, WA 98109, USA; 2 Fred Hutchinson Cancer Center Research, Program in Computational Biology, Division of Public Health Sciences, Fairview Avenue North, Seattle, WA 98109, USA; 3 INSERM, IRISA Symbiose, Campus de Beaulieu, 35042 Rennes Cedex, France; 4 Automated Biotechnology, Merck & Co, North Wales, PA, 19454, USA

## Abstract

**Background:**

A systems biology interpretation of genome-scale RNA interference (RNAi) experiments is complicated by scope, experimental variability and network signaling robustness. Over representation approaches (ORA), such as the Hypergeometric or z-score, are an established statistical framework used to associate RNA interference effectors to biologically annotated gene sets or pathways. These methods, however, do not directly take advantage of our growing understanding of the interactome. Furthermore, these methods can miss partial pathway activation and may be biased by protein complexes. Here we present a novel ORA, protein interaction permutation analysis (PIPA), that takes advantage of canonical pathways and established protein interactions to identify pathways enriched for protein interactions connecting RNAi hits.

**Results:**

We use PIPA to analyze genome-scale siRNA cell growth screens performed in HeLa and TOV cell lines. First we show that interacting gene pair siRNA hits are more reproducible than single gene hits. Using protein interactions, PIPA identifies enriched pathways not found using the standard Hypergeometric analysis including the FAK *cytoskeletal remodeling pathway*. Different branches of the *FAK *pathway are distinctly essential in HeLa versus TOV cell lines while other portions are uneffected by siRNA perturbations. Enriched hits belong to protein interactions associated with cell cycle regulation, anti-apoptosis, and signal transduction.

**Conclusion:**

PIPA provides an analytical framework to interpret siRNA screen data by merging biologically annotated gene sets with the human interactome. As a result we identify pathways and signaling hypotheses that are statistically enriched to effect cell growth in human cell lines. This method provides a complementary approach to standard gene set enrichment that utilizes the additional knowledge of specific interactions within biological gene sets.

## Background

The ability to study a gene's contribution to phenotype through RNA interference (RNAi) has provided unprecedented insight to the essential biology of mammalian cell lines. RNAi knockdowns inhibit messenger RNA translation leading to changes in protein concentration, protein interactions, transcription, and ultimately an effect on phenotype [1-3]. Genome-scale siRNA phenotype screens consist of thousands of targeted perturbation experiments to identify significant effectors on a phenotype of interest, such as cell growth. As these high-throughput screens become more automated and less expensive, there is a growing demand to associate siRNA hits with the interactome.

Unfortunately, the interpretation of genome-scale RNAi phenotype screens is complicated by several sources of experimental variability. Off-target effects arise when the change in phenotype is not a result of a targeted mRNA knockdown, but rather the knockdown of some other mRNA. Cell-line specific differences in RNAi efficacy may result in attenuated knockdown phenotypes for essential effector genes [4-6]. Furthermore, the robustness of genetic regulatory networks complicates the analysis of RNAi phenotype data. Gene knockout studies have demonstrated that a minority of genes, only 19% in *S. cerevisiae*, are lethal when deleted under laboratory growth conditions [7]. Genome-scale knockdown studies in *Drosophila *and human cell lines also demonstrate that a relatively small proportion of knockdowns affect growth phenotypes [8,9]. Several reasons for robustness include signaling modularity, redundancy and feedback loops [2,10-12]. As a result, knockdowns that cause an impaired growth phenotype provide a glimpse to uncommonly sensitive areas of cell signaling.

Gene set enrichment methods are a conventional tool in the analysis of high throughput datasets. These established statistical protocols were originally used to associate differentially expressed genes from microarray experiments with biologically annotated gene sets such as Gene Ontology (GO) categories, canonical pathways, or protein complexes [5,8,13-16]. These over representation approaches (ORA) use a statistic, such as Hypergeometric or average z-score, to assign a p-value that is the probability of seeing the observed overlap of a gene hit list and gene set by chance. ORA methods, however, do not directly take interactions between specific set members into account and this is additional biological information that can be utilized in knowledge-based enrichment approaches. For example, the EGFR pathway contains four types of ErbB family tyrosine kinase receptors that are activated by distinct ligands (e.g. EGF, TGFα) and initiate distinct signal transduction cascades [17]. Consequently, the specific combination of screen hits represented in a pathway provides additional information beyond the simple count of hits occurring in this pathway. An ORA that takes advantage of known connectivity between gene set members provides a complementary view to the results provided by conventional enrichment methods (i.e. the Hypergeometric) and identify signaling events that are enriched for siRNA hits.

To our knowledge, the only pathway enrichment method that takes advantage of knowledge of specific interactions within gene sets was presented by Draghici *et al. *to analyze gene expression signatures[18]. An impact analysis is used to count all possible paths (interactions) between differentially expressed genes in KEGG pathways. Unfortunately the pathway score is weighted by classic Hypergeometric enrichment analysis (HGA) and the authors do not discuss how results differ based solely on intra-pathway connectivity. This method is also subject to connectivity biases of each gene product causing highly connected genes to be counted in more paths.

Several other papers have interpreted RNAi data using protein interactions. Work by Friedman *et al. *combined an RNAi screen assayed by protein readout of extra-cellular regulating kinase (ERK) with literature-curated protein interactions to produce a protein interaction network [4]. Another approach recently published by Huang *et al. *uses gene set enrichment of GO categories to select hits and then connects them with literature-reported protein interactions [19]. The interactome is known to be highly-connected so it is not surprising that protein interactions are found between hits. These approaches do not take into account difference in connectivity between gene products. This bias is cause for concern because knockdown gene hits that are involved in many annotated protein interactions are more likely to be connected with other hits simply by chance.

We propose here an ORA method called protein interaction permutation analysis (PIPA) that takes advantage of literature-curated protein interactions between gene products within gene sets. This method uses a graph permutation algorithm to create a null distribution that takes into account connectivity biases of the known interactome to identify genet sets with statistically enriched interactions between RNAi targeted gene products. It is our hypothesis that pathways enriched for interactions connecting RNAi hits effecting cell growth capitulate essential signaling.

We use PIPA to analyze siRNA cell growth phenotype screens performed on HeLa and TOV cell lines. To justify using a gene interaction ORA, we show that hits mapping to interacting gene pairs are more reproducible between replicate screens. Next we show that PIPA uniquely identifies statistically enriched pathways that the Hypergeometric does not. Finally we produce a global essential signaling network using enriched protein interactions and annotate portions of this network using GO categories.

## Results and Discussion

### Protein Interaction Permutation Analysis (PIPA) Algorithm

For a given gene set, PIPA identifies the probability of seeing an observed number of protein interactions between siRNA hits by chance. We start with a gene set derived from a GeneGo Metacore Pathway. This gene set, *G*, is filtered to only contain genes targeted in the siRNA screen. Gene set members are labeled as "hits" or "non-hits" as described in the methods section. Gene set members are connected using literature-curated interactions to create a network *N*_*G *_where genes are represented as nodes and interactions are represented as edges. We label the number of observed edges connecting hits as *O*.

A network permutation method is used to derive a null distribution by permuting node labels. This approach is an extension of the graph permutation algorithm presented by Balasubramanian *et al. *and allows the topology of *N*_*G *_to remain constant [20]. By shuffling node labels we sidestep the connection bias of highly annotated (highly connected) gene set members. A minority of the knockdown genes in our siRNA screen are identified as hits (~6%) and accordingly a minority of permutations will label these highly connected nodes as hits.

Starting with network *N*_*G*_, we shuffle node labels with all other genes targeted in the screen to create a shuffled version of *N*_*G *_we refer to as . The number of edges connecting hits within  is recorded. This process is iterated 10,000 times and a p-value is calculated as the proportion of sampled permutations that have *O *or greater edges connecting hits. We use a p-value threshold of 0.05 to reject the null hypothesis that the number of observed edges connecting hits is what we would expect by chance. Because of differences in overlap between members and networks across gene sets, we leave p-values unadjusted for multiple testing for a fair comparison between PIPA and conventional hypergeometric enrichment analysis.

We divide the global interactome network into sub-networks that are based on canonical gene sets for several reasons. As an initial experiment, we applied the above permutation algorithm to the global network and found that hits were not significantly connected. However when focusing on sub-networks derived from gene sets, we find significant enrichment for highly connected hits. This finding is consistent with work by Horvath *et al. *where network properties from co-expression sub-networks (modules) are more predictive than network properties from the global co-expression network [21]. Another advantage to dividing the global interactome into gene sets, is that by their definition canonical gene sets are biologically annotated and provide an intrinsic systems biology explanation of hits. Finally, by focusing on these sub-networks we are able to divide-and-conquer the global network in a biologically supported and computationally tractable manner.

### Interacting gene pair siRNA hits are more reproducible than single gene hits

**Figure 1:**
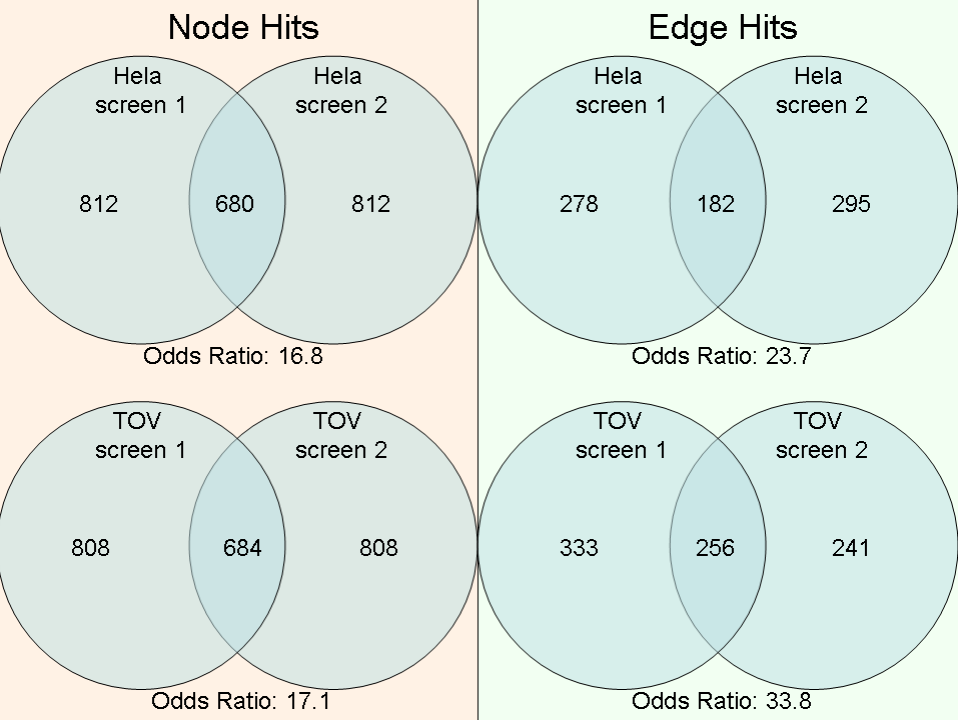
**"Node hits" represent all single gene siRNA hits while "edge hits" represent siRNA hits connected by a literature-curated protein interactions from Metacore, HPRD, and/or Ingenuity**. Higher odds ratios for edge hits (right) show a stronger overlap across replicate HeLa and TOV screens than node hits (left). Odds ratios calculated with universe sizes 18,586 and 11,426 for node hits and edge hits respectively.

While replicate screens ideally should result in identical hit lists, experimental variability makes this not the case. A guiding principle behind ORA approaches is hits from pathways enriched for hits are more likely to be true positives and represent relevant biological processes. Similarly, a guiding hypothesis behind the development of PIPA is that screen hits that are directly connected in the global interactome are more likely to be true positives than are individual hits. To test this hypothesis we compared the overlap of single gene screen hits (node hits) to the overlap of interacting gene pair siRNA hits (edge hits) from replicate screens carried out in HeLa and TOV cell lines (Figure 1). Experimental description, data pre-processing, and labeling of hits are detailed in the Methods section. While significant overlap is observed for node hits in both screens, the overlap between edge hits indeed shows greater statistical significance.

Of note, the requirement that two hits share an interaction substantially reduces the number of edge hits compared to node hits. If we view nodes that are connected by edges as random independent variables with a ~6% chance of being a hit, the likelihood of drawing two hits by chance (~0.36%) is dramatically smaller. The use of interaction data to select among hits, consequently, substantially reduces the total number of hits while simultaneously enriching for hits that are more likely to be reproduced in replicate screens. This observation supports our hypothesis that analyzing high-throughput siRNA data in the context of protein interactions is likely to enable identification of essential signaling cascades.

### PIPA identifies both protein complexes and signaling interactions

**Figure 2:**
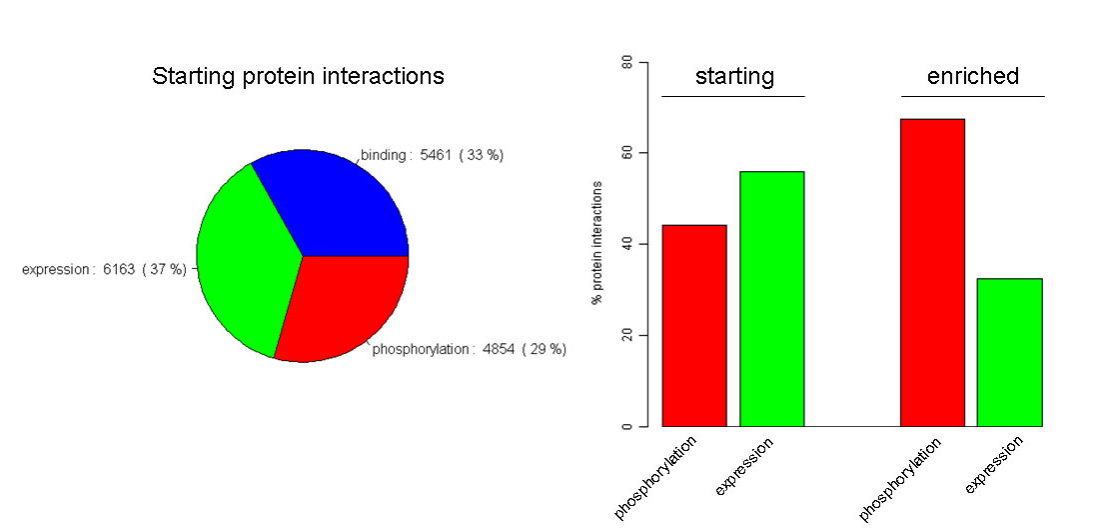
**The integrated interactome data set is comprised of 16,478 protein interactions, roughly evenly distributed between binding, phosphorylation and expression regulation (left)**. Phosphorylation and expression regulation interactions (11,017 total) were used to identify enriched gene sets in HeLa and TOV genome wide siRNA screens using PIPA. The edge hits from enriched gene sets (right) are enriched for phosphorylation interactions.

To utilize gene interaction information in combination with gene sets, the PIPA algorithm identifies gene sets in which edge hits are statistically overrepresented compared to a null distribution obtained by shuffling the node labels in each gene set. In the analysis of the HeLa and TOV screening data, we identified enriched pathways by combining GeneGO Metacore canonical signaling pathways with a human interactome map comprised of the union of binding, phosphorylation and expression regulation edges from three data sources (see Methods). The intersection of this interactome map with the canonical pathway gene sets yields 16,478 interactions, roughly evenly distributed among the three interaction types (Figure 2).

When PIPA is carried out using all 16,478 protein interactions, the majority of edge hits in enriched gene sets result from binding interactions (86.9%). Of these enriched binding edges, 72.5% are from protein complexes, as defined by GO Cellular Component categories that are children of the "protein complex" category (GO:0043234). It is expected that sub-units of protein complexes will have similar phenotypes in RNAi screens, and in fact RNAi studies often use essential protein complexes to identify false negatives because each knockdown targeted complex sub-unit is known to have a significant effect on phenotype [9,15]. Additionally protein interaction databases typically include binding interactions between each sub-unit of large complexes, for example the eukaryotic initiation factor 3 (EIF3) or proteasome, resulting in highly-connected graphs between 13 or 27 complex sub-units (respectively) [22]. As a result, any edge-based ORA will be biased by these highly-connected portions of the global interactome map.

Although the identification of interactions between sub-units of essential protein complexes represents true biology, these highly connected pieces of the interactome obscure regulatory protein interactions which are of more interest for understanding cell signaling. Consequently, to identify essential signaling cascades, we restricted the interaction data set to 11,017 phosphorylation and expression regulation interactions. With this signaling-focused interaction set, 80 edge hits in enriched pathways were identified using PIPA. Of note, while the initial interaction set contained roughly equal proportions of phosphorylation and expression regulation edges, the edge hits from the enriched pathways were significantly enriched for phosphorylation interactions (Figure 2), additionally supporting the hypothesis that PIPA is detecting essential signaling events. Interestingly, this result also suggests that expression regulation is more robust to perturbation than is phosphorylation. This observation may reflect the fact that expression regulation interactions reported in these databases are more likely to be indirect interactions mediated by other genes and gene products than are phosphorylation interactions.

### PIPA identifies distinct enriched pathways relative to the Hypergeometric

**Figure 3:**
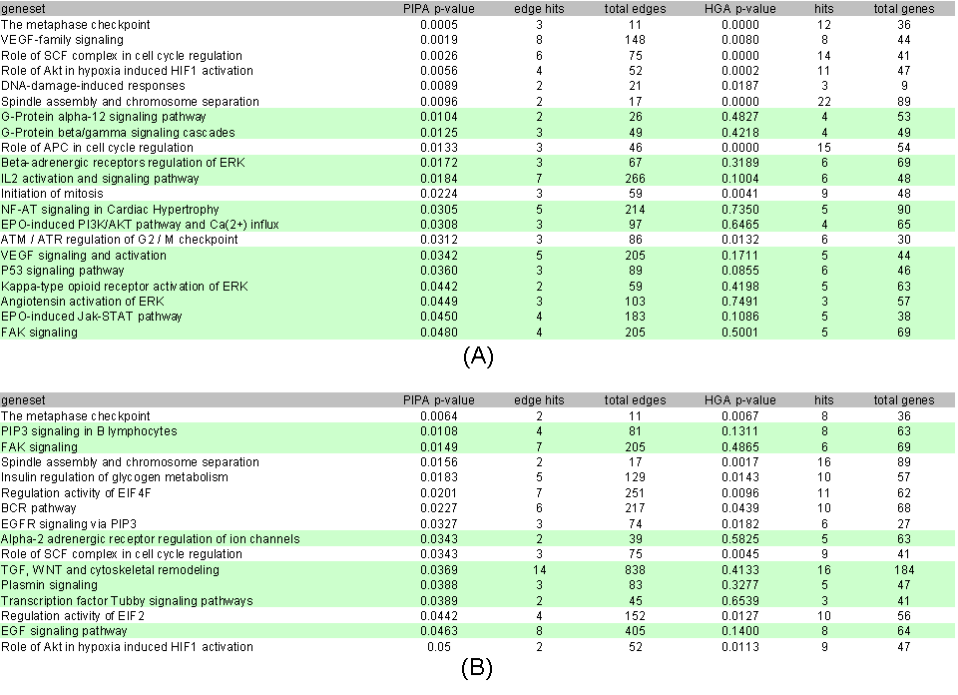
**Enriched pathways identified using PIPA for (A) HeLa and (B) TOV siRNA hits are shown**. Enriched pathways uniquely identified using PIPA are highlighted in green.

We applied PIPA to the HeLa and TOV siRNA screens to identify pathways that show significant enrichment for protein interactions connecting siRNA hits (Figure 3). Pathways with expected biological relevance to cell cycle regulation, translation, cell structure and various signal transduction cascades are shown to be enriched. Five enriched pathways are common to both HeLa and TOV cell lines: metaphase checkpoint, spindle assembly and chromosome separation, cytoskeletal remodeling focal adhesion kinase (FAK) signaling, role of SCF complex in cell cycle regulation, and role of Akt in hypoxia induced HIF1 activation.

**Figure 4:**
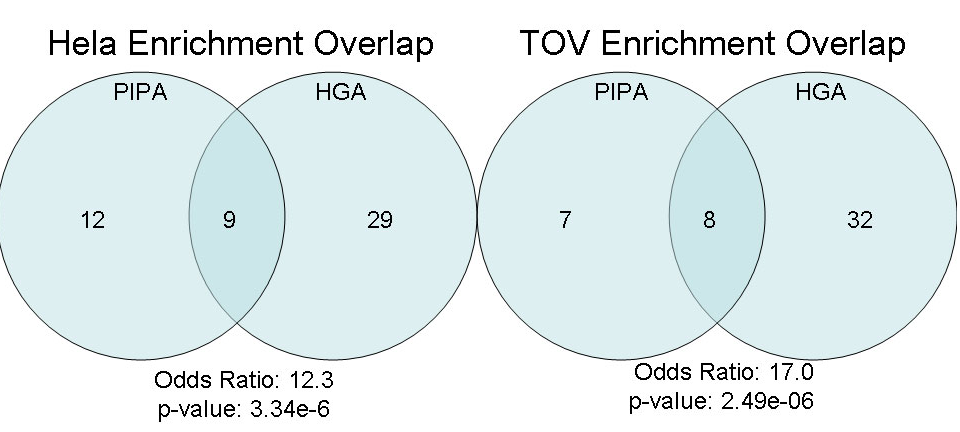
**Venn diagrams indicate the number of overlapping and unique enriched pathways identified by using PIPA and Hypergeometric analysis (HGA) methods**. While there is strong agreement, nearly half of the PIPA enriched pathways are not found using HGA.

PIPA identifies a distinct set of enriched pathways relative to HGA (Figure 4). Odds ratios and p-values indicate a strong agreement between methods, but approximately half of the enriched gene sets identified by PIPA are not detected by HGA (see Figure 3). Of note, because the coverage of the interactome is incomplete, many pathways have sparse interaction coverage, leading to fewer enriched gene sets detected by PIPA relative to HGA. As annotation and completeness of the interactome progresses, this limitation of edge-based approaches such as PIPA is expected to diminish.

### PIPA detects active signaling branches with pathway gene sets

**Figure 5:**
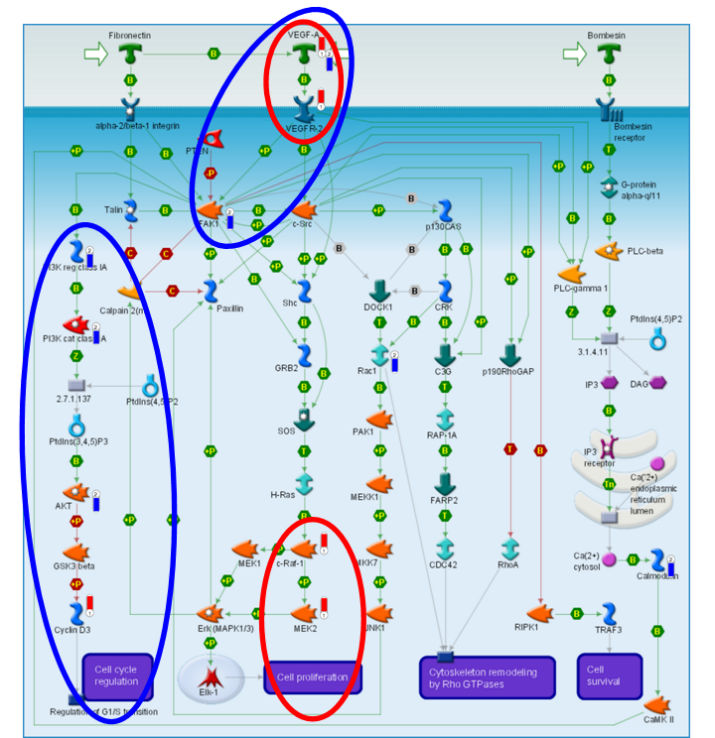
**The Cytoskeletal Remodeling: FAK Signaling pathway is enriched for HeLa and TOV edge hits using PIPA but not node hits using HGA enrichment**. Red indicators are HeLa siRNA hits and blue indicators are TOV hits. Both HeLa and TOV show significant enrichment of protein interactions connecting hits (p-value = 0.048 and p-value = 0.015 respectively). Different signaling branches in this pathway have lethal effects in the two different cell lines when perturbed, although both branches are annotated as associated with a cell growth phenotype. [GeneGo Metacore (2008)].

The *Cytoskeletal Remodeling: FAK Signaling *pathway (Figure 5) is significantly enriched for both HeLa and TOV siRNA hits using PIPA and not significantly enriched using HGA. FAK-mediated signal transduction has been implicated in control of cell migration, cell cycle progression and apoptosis [23]. Interestingly, both cell lines showed the VEGFA growth factor as being a hit while different branches of the pathway showed enrichment for edge hits. TOV hits highlight hits on FAK, PI3K and Akt signaling whereas HeLa hits show enrichment of MAPK signaling via Raf1 and Mek2. PIPA identifies enrichment in this pathway using protein interactions despite their being few scattered hits on the right side. Our results indicate that bombesian receptor signaling is not essential to HeLa and TOV cell lines.

### Essential Signaling Network Identified in HeLa and TOV screens

**Figure 6:**
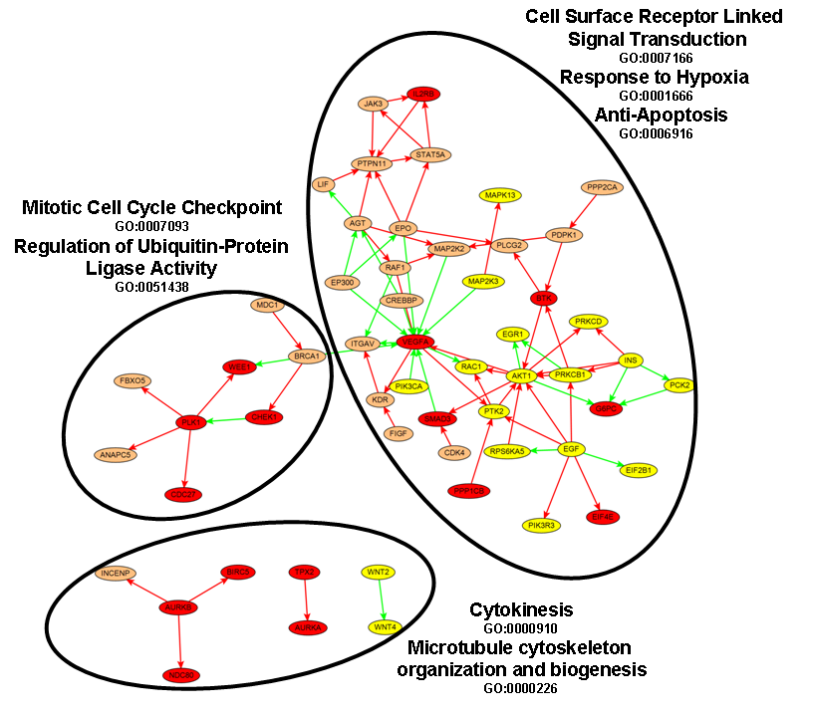
**Combined edge hits from PIPA enriched gene sets from HeLa and TOV screens**. The three major clusters show enrichment for a variety of biological functions that allow cells to maintain cytoskeletal organization, proteolysis, and prevent apoptosis. The indicated GO Biological Process categories were shown to be significantly enriched after Bonferroni correction. Orange nodes are HeLa hits, yellow nodes are TOV hits, and red nodes are hits in both screens. Edge color indicates the type of protein interaction (red = phosphorylation, green = expression regulation). GO category enrichments were calculated using the Hypergeometric and p-values (< .001) are Bonferroni corrected.

To identify a sub network of essential signaling interactions detected in the siRNA screens, edge hits from gene sets enriched using HeLa and TOV hits were combined into a single large network (Figure 6). After visualizing the network using a force-based clustering algorithm, major clusters pertaining to GO categories relevant to cell growth rate can be identified. Of note, starting with the original hit list, no enriched GO terms are identified by HGA. Application of PIPA, as a result enables core signaling interactions and processes to be identified from comparatively noisy genome-wide siRNA screening data.

## Conclusion

Genome scale siRNA screens provide an exciting opportunity to investigate the interactome. One caveat of these high-throughput screens is that generally only a few cell attributes are measured after each perturbation. There is a strong need to map hits to the interactome for biological interpretation and generation of signaling hypotheses. Here we present an ORA that incorporates protein interactions to identify canonical signaling pathways enriched for siRNA hits. This method extends and complements traditional gene set enrichment methods by specifically incorporating prior knowledge about the interactions within a pathway using a graph-based permutation algorithm. Also, PIPA provides an enrichment framework to exclusively examine signaling events within canonical pathways without the biases introduced by highly-connected multi-subunit complexes. When applied to genome wide siRNA screens in HeLa and TOV cell lines, the edge hits in identified pathways are enriched for GO categories relevant to the cell cycle, anti-apoptosis, and cytoskeletal structure. Of note, different branches of the *FAK Signaling *pathway are specifically effected by siRNA perturbations in HeLa versus TOV cells lines.

Protein interaction databases provide an initial view of the human interactome that is known to be incomplete [24]. In addition, there is no universal standard for defining and annotating interactions across databases leading to differences in interaction accuracy. Also, the contextual information about interactions is generally absent from such databases, making it difficult to identify coherent sets of interactions that constitute functional signaling networks. These limitations in the known interactome inevitably lead to limitations in any methods relying on interactome data. Here we combine interactome interactions with canonical gene sets to partially address some of these limitations. As the quality of interactome databases increase, it is expected that the increased coverage and contextual information will be reflected in the improved ability of edge-based enrichment methods, including PIPA, to identify biologically meaningful signaling modules.

The process of natural selection is present in human tumors and a driving force behind cancer treatment resistance [25]. Thus, it is critical to understand what pathways and protein interactions are sensitive to perturbations in a given interactome. Pathways enriched for interactions connecting siRNA hits make available an exciting portrait of essential signaling that is cell line specific and has the potential to guide future treatment strategies.

## Methods

### Genome-scale siRNA screens

Genome-scale siRNA knockdown screens were performed in duplicate on HeLa and TOV cell lines essentially as described before [26]. The siRNA library is comprised of 18,586 unique siRNA pools, where each pool is an equimolar mixture of three siRNAs targeting different sequences of the same mRNA transcript. Both Hela and TOV21G cells were cultured in Dulbecco's Modified Essential Medium (DMEM) supplemented with 10% fetal bovine serum and penicillin/streptomycin. For the assay, 400 cells/well (Hela) or 1000 cells/well (TOV21G) in DMEM were seeded in wells of a 384-well TC-treated microplate. 24 hrs after cell plating, diluted Oligofectamine (1:40 in Opti-MEM) was incubated with siRNA for 20–30 min, and cells were transfected with the Oligofecatmine/siRNA mixture. Final concentration of siRNA pools was 50 nM and 25 nM for Hela and TOV21G screens, respectively. 72 hours post-transfection, cell viability was measured by Alamar Blue fluorescence assay. An siRNA to luciferase was used as a negative control (100% viability) and a no cell control was used a 0% viability control. A siRNA to Polo-like kinase 1 (Plk1) was used as a positive biological control to monitor transfection efficiency throughout the screens.

Cell growth phenotype values from siRNA screens were converted to z-scores, and quantile normalization was performed across all four screens to make a z-score hit threshold comparable across screens. Note that normalization steps result in the same number of node hits for replicate screens shown in Figure 1. For enrichment analyses, z-scores for each knockdown in duplicate screens were averaged together within each cell line. Averaged z-scores less than -1.5 were labeled as siRNA hits having a strong negative effect on cell growth. This threshold is based on other published genome-scale RNAi analyses and chosen to include expected essential genes (PLK1, KIF11, ARPC3) [4,27-29]. In our enrichment analysis, 1,133 (6.1%) HeLa and 1,108 (6.0%) TOV knockdowns meet this criterion and are labeled as hits. Results shown in Figure 1 comparing replicate screens are non-averaged z-scores using the same z-score threshold to label hits.

### Interactome Data Set and Canonical Gene Sets

Literature-curated interactions from GeneGo, Inc., Ingenuity^®^, and the Human Protein Reference Database (HPRD) were combined and filtered to be unique, non-self, and non-reciprocal [30-32]. There were 98,561 annotated interactions pertaining to phosphorylation, gene expression and physical binding events that mapped to siRNA targeted genes. We focused on these three interaction types as they were universal between all three protein interaction databases and made up the majority of interactions. GeneGo's Metacore canonical pathways (524 total) were used to provide contextual information to the global interactome map by grouping genes and/or gene products into sets that function together in a canonical biological processes. The intersection of the global interactome map and each canonical gene set was used to approximate topologically well-defined signaling pathways.

### Overlap effect size calculations

Overlap effect size is estimated by computing the odds ratio on a 2 × 2 table obtained by classifying the relations of replicate screens. The edge hit universe (11,426) is calculated by counting the number of unique siRNA knockdowns belonging to protein interactions where both interacting partners are targeted in the screen. The edge hit overlap is calculated by taking the intersection of hits belonging to protein interactions connecting two siRNA hits.

## Abbreviations

GO: Gene Ontology; RNAi: RNA interference; siRNA: small interfering RNA; ORA: over-representation approaches; PIPA: protein interaction permutation analysis; HPRD: human protein reference database; FAK: focal adhesion kinase; HGA: Hypergeometric enrichment analysis.

## Authors' contributions

AB designed and implemented the method and analysis. CR and RG were critical to method design and data analysis. AB and CR drafted the manuscript. IS collected protein interactions and gene sets. IS and MK developed network visualization software. CN contributed to display of results, method name, and valuable discussion. NLM contributed to method design. MF oversaw siRNA screen data collection and wrote methods section describing data collection. All authors read and approved the final manuscript.
